# A survey on the management of chronic myeloid leukemia in chronic phase from the perspectives of both patients and physicians

**DOI:** 10.1007/s00277-026-07103-z

**Published:** 2026-06-16

**Authors:** Ahmet Emre Eşkazan, Samet Yaman, Abdulkerim Yıldız, Deniz Özmen, Emel Gürkan, Ünal Ataş, Nevin Alayvaz Aslan, Özgür Mehtap, Selim Sayın, Seda Yılmaz, Ayşe Uysal, Mehmet Fatih Orhan, İbrahim Ethem Pınar, Nur Soyer, Mehmet Baysal, Özlem Gür, Barış Uçar, Mehmet Yılmaz

**Affiliations:** 1 Division of Hematology, Department of Internal Medicine, Cerrahpaşa Faculty of Medicine, Istanbul University-Cerrahpaşa, Istanbul, Türkiye; 2 Division of Hematology, Department of Internal Medicine, Faculty of Medicine, Hitit University, Çorum, Türkiye; 3 Division of Hematology, Department of Internal Medicine, Faculty of Medicine, Çukurova University, Adana, Türkiye; 4 Division of Hematology, Alanya Training and Research Hospital, Antalya, Türkiye; 5 Division of Hematology, Department of Internal Medicine, Faculty of Medicine, Pamukkale University, Denizli, Türkiye; 6 Division of Hematology, Department of Internal Medicine, Faculty of Medicine, Kocaeli University, Kocaeli, Türkiye; 7 Division of Hematology, Department of Internal Medicine, Gülhane Training and Research Hospital, Ankara, Türkiye; 8 Division of Hematology, Konya City Hospital, Konya, Türkiye; 9 Division of Hematology, Department of Internal Medicine, Fırat University, Elazığ, Türkiye; 10 Division of Pediatric Hematology and Oncology, Department of Pediatrics, Faculty of Medicine, Sakarya University, Sakarya, Türkiye; 11 Division of Hematology, Isparta City Hospital, Isparta, Türkiye; 12 Division of Hematology, Department of Internal Medicine, Faculty of Medicine, Ege University, Izmir, Türkiye; 13 Division of Hematology, Department of Internal Medicine, Faculty of Medicine, Tekirdağ Namık Kemal University, Tekirdağ, Türkiye; 14 Division of Medical Affairs, Novartis Pharmaceuticals Corporation, Istanbul, Türkiye; 15 Division of Hematology, Department of Internal Medicine, Faculty of Medicine, SANKO University, Gaziantep, Türkiye

**Keywords:** Chronic myeloid leukemia, Survey, Disease management, Patient perspective, Physician perspective

## Abstract

The lifespan of patients with chronic phase chronic myeloid leukemia undergoing tyrosine kinase inhibitor treatment has nearly reached that of the general population. However, treatment switching due to resistance or intolerance demonstrates the significance of effective communication between patients and physicians in decision-making. This study aimed to evaluate priorities, expectations, and perceptions of managing CML-CP from the perspective of both patients and physicians in Türkiye. This cross-sectional survey study was conducted from July to December 2024 by the Turkish Society of Hematology. A total of 129 hematologists completed a web-based survey, while 120 patients receiving TKI treatment completed a structured telephone survey. Eleven common questions were analyzed descriptively to compare perspectives. Patients (53% male; median age 54 years) and physicians agreed on essential information to be communicated at treatment start, although priorities differed. Patients prioritized information about disease progression, daily life impact, and treatment effectiveness, whereas physicians focused on treatment safety, patient monitoring, and follow-up assessments. Patients (40.8%) emphasized treatment effectiveness when switching therapies, while physicians (46.7%) focused on the manageability of adverse events. Approximately 25% of patients reported suboptimal adherence, mostly due to forgetfulness (52%). Follow-up visits occurred every 2 to 3 months, with a mean duration of approximately 10 min. Although patients and physicians shared broadly similar views, discrepancies existed in their prioritization of factors and overall opinions. Patients expressed greater optimism regarding the disease impact on their daily lives. Enhancing communication between patients and physicians, as well as promoting shared decision-making during treatment transitions, may improve adherence and disease management outcomes.

**Supplementary Information:**

The online version contains supplementary material available at https://doi.org/10.1007/s00277-026-07103-z.

## Introduction

Chronic myeloid leukemia (CML) is a clonal myeloproliferative neoplasm characterized by the presence of the Philadelphia chromosome. Life expectancy in CML in chronic phase (CML-CP) has become close to that of the general population with tyrosine kinase inhibitors (TKIs) [1]. The availability of multiple first-, second-, and third-generation TKIs has enabled individualized treatment strategies based on efficacy, safety, comorbidities, and patient preferences. Currently, four TKIs are authorized for the treatment of CML in Türkiye: imatinib, nilotinib, bosutinib, and dasatinib. TKI switching is often necessitated in clinical practice, especially due to drug resistance and intolerance [2–5]. Despite these therapeutic advances, CML management still involves significant clinical and practical challenges.

The importance of treatment tolerability, adherence, and quality of life in decision-making has been underscored by real-world studies that have shown TKI switching is a common occurrence in routine clinical practice, which is most frequently driven by intolerance and, less commonly, by resistance [2, 5]. Besides, treatment goals in CML-CP have evolved beyond survival to include achieving deep molecular responses, maintaining long-term tolerability, and, for selected patients, achieving treatment-free remission (TFR) [1].

There is an increasing emphasis on patient-centered care, which has highlighted the necessity of understanding patients’ perspectives, expectations, and concerns related to treatment. A multinational survey, the Chronic Myeloid Leukemia – Study of Unmet Needs (CML-SUN) was conducted across 11 countries. The data collected from that study have provided valuable insights into patient and physician priorities and treatment goals in CML [6]. The study revealed that patients prioritize prevention or slowing disease progression, maintenance or improvement of quality of life, and management of adverse events, while physicians focus on treatment efficacy. Patient participation in treatment decision-making was limited: only 19% to 26% reported discussing treatment options and anticipated outcomes with their physicians. Conversely, 44% to 48% of hematologists reported making treatment decisions independently, with minimal or no patient involvement [6]. These findings highlight the significance of effective communication and collaborative decision-making to better address unmet expectations within CML management.

Real-world studies from Türkiye have shown diverse patient characteristics, treatment patterns, and clinical management practices among patients with CML, indicating variability in standard care across centers [3]. The incidence of CML in Türkiye was reported as approximately 0.9/100.000 adults and the prevalence was approximate 8/100.000 inhabitants in Türkiye [4]. However, these studies have mostly focused on clinical outcomes and treatment patterns, while data assessing patient and physician perspectives, decision-making processes, treatment expectations, and priorities is still limited. To the best of our knowledge, no study in Türkiye has evaluated treatment goals and expectations in CML-CP simultaneously from both patient and physician perspectives.

Therefore, this study aimed to evaluate diagnostic, treatment, and follow-up processes in CML-CP from both patient and treating physician perspectives, with a particular focus on treatment decision-making, concerns related to the disease and its treatment, as well as treatment goals and expectations. By identifying unmet needs in CML-CP management, this study aims to contribute to the development of practical solutions that can be implemented in clinical practice to better address expectations and further improve clinical outcomes.

## Methods

### Study design

This national, cross-sectional, descriptive survey study assessed treatment goals, anticipations, and management strategies in CML-CP from the perspective of both patients and physicians. The study was carried out from July 2024 to December 2024 in collaboration with the Turkish Society of Hematology Chronic Myeloproliferative Neoplasms Scientific Subcommittee. Two questionnaires were answered by hematology physicians and CML-CP patients receiving TKI treatment.

### Survey

Both questionnaires were based on CML-SUN survey instruments and modified to meet the objectives of this study by Panacea Research using Alchemer web-based survey platform. The study team and the corresponding author reviewed and revised the questionnaires for their relevance to the Turkish clinical setting and study objectives. Pilot testing was undertaken independently prior to the study for both the patient and physician questionnaires, with three pilot interviews or surveys for each. These pilot tests were conducted to assess the clarity, comprehensibility, structure of the questionnaire, and the application of the Likert-scale questions. The questionnaires included single- and multiple-choice, ranking, and open-ended questions. A total of 129 hematologists across Türkiye who volunteered to participate and fulfilled the eligibility criteria accessed the survey through the official website of the Turkish Society of Hematology (www.thd.org.tr) and completed it online. The physician survey consisted of 29 questions about physicians’ professional background, their perceptions, and personal practices regarding CML diagnosis, management (treatment decision, switch, follow-up, monitoring), treatment goals, and the impact of CML on patients’ lives.

Patients who were found to be eligible to participate were identified and recommended by their treating hematologists during routine clinical visits. Among the hematologists who participated in the physician survey, 15 voluntarily invited eligible patients to take part in the patient survey. The patient survey included 36 questions covering demographic characteristics, diagnosis process, information sharing, decision-making, treatment and switching, disease monitoring, disease perception, and the impact of CML on their lives. A total of 120 patients (≥ 18 years), treated by 15 participating hematologists, completed the survey via telephone interviews conducted by trained surveyors from a designated research company. This recruitment approach may have introduced selection bias, potentially leading to the overrepresentation of patients with stronger physician–patient relationships, better adherence, or greater engagement in their care. The assessment of patients’ perspectives regarding the effect of CML-CP and its treatment on daily life, alongside physicians’ perceptions of CML’s impact on patients, was conducted through structured questionnaires utilizing a 7-point Likert scale (1 = strongly disagree to 7 = strongly agree). The collected data were analyzed anonymously. Additionally, responses to 11 common questions were analyzed descriptively compared between the patients (*n* = 120) and a subgroup of hematologists (*n* = 15) who were treating the surveyed patients. These physicians identified and referred eligible patients voluntarily during clinical practice. The comparison between physicians and patients was conducted at the group level, without utilizing paired matching of individual physician–patient pairs.

### Study participants

Eligible patients were adults (≥ 18 years) who had been receiving TKI therapy for CML-CP for at least one year, regardless of treatment line, and who were literate and able to verbally respond to the survey questions during a telephone interview. Hematology physicians who have been responsible for treatment decisions for at least five CML-CP patients within the past 12 months were included in the study. Physicians and patients who did not provide consent to participate in the study were excluded. No patients or physicians received any remuneration for completing the surveys.

Sample size calculation was performed using the Raosoft program. Based on an estimated population of approximately 6000 patients with CML-CP and 400 hematology physicians in Türkiye, with a Type I error rate of 5% (α = 0.05) and an acceptable margin of error of 10% (d = 0.10), the minimum required sample size was calculated as 105 CML-CP patients and 86 hematology physicians, assuming 10% missing data.

### Ethics approval and informed consent

The study was conducted in accordance with the principles of the Declaration of Helsinki and applicable national regulations. All patients provided written informed consent. Ethics committee approval was obtained from Istanbul University-Cerrahpaşa Ethics Committee (the date: 11 June 2024 and the number:736).

### Statistical analysis

Statistical analyses were conducted using SPSS software. All survey data were analyzed descriptively in accordance with the study design. Categorical variables were summarized using frequencies and percentages, while continuous variables were summarized using means, medians, and interquartile ranges (Q1–Q3), as appropriate. No inferential statistical comparisons were performed.

## Results

A total of 120 patients with CML-CP and 129 hematology physicians were included in the study. 53% of participants were male, and the median age was 54 years (Q1-Q3: 42–63 years). The median time since CML diagnosis was 7 years (Q1-Q3: 3–12 years) with a median current TKI treatment duration of 48 months (Q1-Q3: 24–84 months) (Table 1). The most commonly reported comorbidities were cardiovascular disorders (30%), diabetes (14%), and respiratory diseases (10%).

**Table 1:** Demographic and Clinical Characteristics of Patients With Chronic Phase Chronic Myeloid Leukemia

Patient Characteristics (*n* = 120)	Value
Median age, years (Q1-Q3)	54 (42–63)
Male, % of patients	53
Median time since CML diagnosis, years (Q1-Q3)	7 (3–12)
Comorbidities (top 3), % of patients	
Cardiovascular Diseases	30
Diabetes	14
Respiratory Diseases	10
Employment Status, %	
Retired	30.8
Employed	30.0
Homemaker	25.0
Unable to work due to disease/disability	1.7
Employment status changes due to CML, %	
Not changed	86.7
Temporarily changed/reduced workload	6.7
Early retired	5.8
Lost job	0.8
Patients live with the family, %	94.9
Education Level, %	
Primary school	38.3
High school (including vocational high school)	25.6
University	16.7
Treatment, %	
Primary line	41
Second line	31
Third line or more	28
Median duration of current TKI treatment, months (Q1-Q3)*	48 (24–84)

*Based on available data from 113 patients

Retirees and employees constituted 30.8% and 30% of the study population, respectively, and 1.7% of the participants were unable to work due to CML disease. The majority of patients (86.7%) reported that their working situation remained unchanged after the CML diagnosis, whereas 6.7% reported a temporary work decrease, 5.8% early retirement, and 0.8% job loss. Besides, 94.9% participants were living with family members. Regarding educational status, 38.3% of patients had completed primary school, 25.6% had finished high school, and 16.7% had a university degree.

41% of patients had received first-line therapy, 31% second-line therapy, and 28% third-line or subsequent treatments. Additionally, 21% of participants (*n* = 25) reported missing their medication for a median of 3 days per month (Q1–Q3: 1–4), primarily due to forgetfulness (52%). Of these patients, 20 (80%) said that they informed their physicians about adherence issues. Among the 16 patients with adherence problems who were receiving second-line or later treatments, 10 (63%) reported no change in adherence across treatment lines.

It was reported that 86% of participants had received necessary information from their physicians at diagnosis, and 93% reported they fully understood the treatment information. Among surveyed patients, 59% of patients indicated that they were informed about what to do in case of treatment inefficacy or adverse events, 38% about treatment expectations and evaluation of treatment effectiveness, and 23% about treatment discontinuation under disease control. Physicians were the primary information source, followed by other healthcare professionals (96%) and pharmacists (92%), whereas social media use was limited (10.8%).

At the time of diagnosis, the most common information needs of patients were treatment administration, disease course and quality of life, diagnostic tests and follow-up procedures, and clinical conditions affecting treatment (Table 2). Treatment switch occurred in 71 patients, primarily due to adverse events (43%) and inefficacy (42%). In patients who reported treatment switch due to adverse events (*n* = 31), 97% promptly informed their physicians. Treatment switch was perceived as normal by 35% of patients, whereas 32% reported fear or anxiety. The primary concerns during treatment switch were the new treatment’s ability to control disease progression (41%), fewer side effects (38%), and rapid onset of effect (20%).

**Table 2:** Information Needs at Diagnosis and Priorities During Treatment Switching Among Patients With Chronic Phase Chronic Myeloid Leukemia

Key Information Needs at Diagnosis ^a^	Patients’ opinion, n (%) *N* = 120
The prescribed medication (instructions for use)	117 (98)
The course of CML/ and the impact of CML on daily life	117 (98)
Monitoring and follow-up	110 (92)
Potential side effects of the prescribed medication	106 (88)
Clinical conditions/diseases that may affect therapy	82 (68)
Priorities During Treatment Switching	***n*** (%) N = 71*
Treatment effectiveness (stopping/slowing disease progression)	29 (41)
Management of side effects	27 (38)
Rapid onset of therapeutic effect	14 (20)

^a^ Frequencies represent the proportion of patients selecting each item

^*^ Number of patients who underwent treatment switching

In the entire patient population, 91% of patients had never received psychological support at any time; however, 82% reported that their greatest fear regarding CML was relapses or disease progression. While 90% of patients felt comfortable discussing CML with their physicians, 4% were not, 3% were unsure what to ask, 2% hesitated to ask too many questions, and 1% felt the visit was not long enough for a detailed discussion.

**Fig. 1:**
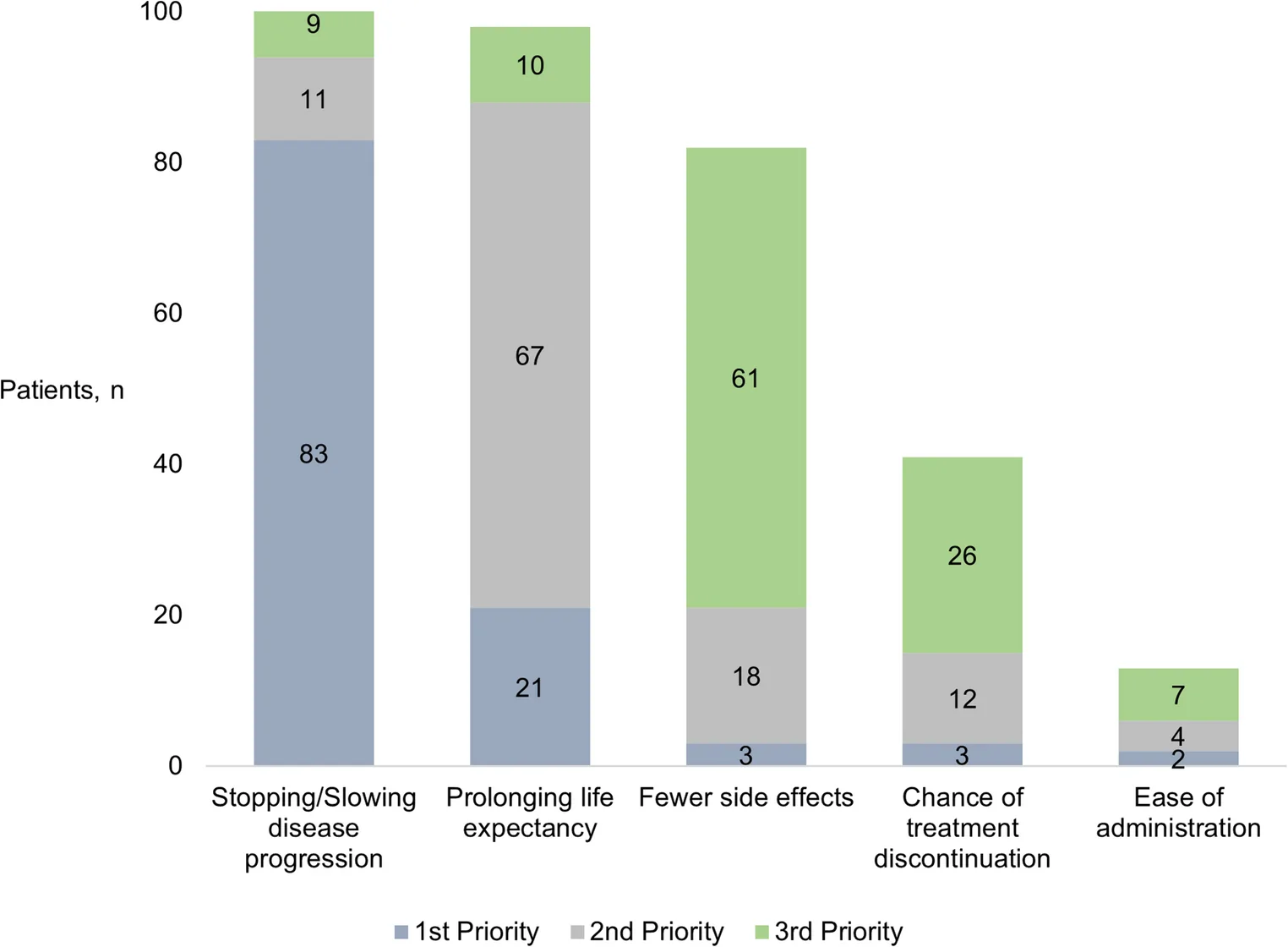
Patient-reported treatment expectations in CML. Percentages were calculated based on available responses for each priority level. Percentages were calculated based on available patient responses for each priority level (*n* = 120)

Disease control results were given priority in patients’ treatment expectations (Fig. 1). The most frequently stated first-priority expectation was to stop or slow disease progression (76.3%), whereas the most widely reported second-priority expectation was to increase life expectancy (43.3%). Expectations for fewer side effects were more commonly expressed among third-priority choices (34.7%). In contrast, ease of administration was mentioned less often at all priority levels.

A total of 129 hematologists from 33 private and public healthcare institutions across different regions of Türkiye participated in the survey, reflecting a geographically diverse cohort. 66% were academicians, and all were actively involved in CML-CP management. The median number of CML patients seen in the past 12 months was 25 (Q1-Q3: 10–50). A median of 80% (Q1-Q3: 50%-90%) of physicians diagnosed their patients themselves (*n* = 127). The mean proportion of patients in the CP was 95% and 71% receiving first-line treatment, 22% second-line treatment, and 7% third-line or later treatment. Imatinib (93.7%) was the most commonly prescribed first-line TKI, while dasatinib was the most frequently used TKI in the second- (46.6%) and third-line (32%) treatments. Although over 90% of participants considered efficacy, safety, comorbidities, and guideline recommendations when selecting treatment, fewer considered risk index and treatment-free remission as important, were 53% and 69%, respectively.

Moreover, 99.2% of physicians stated that clinical trial data were important in treatment decision-making. Among the participating physicians (*n* = 129), while 48% reported hesitation about enrolling patients in clinical studies, 89% believed that they would have access to new treatment options in the future, enabling them to treat their patients better. Physicians most reported providing information on drug usage (98.4%), required tests and follow-up procedures (96%), side effects (95.2%), disease course and impact on daily life (92.8%), and expected treatment outcomes (80%). Among physicians, 49% reported informing patients about various treatment options and involving them in decision-making. However, only 11% believed that patients fully understood the information provided.

**Fig. 2:**
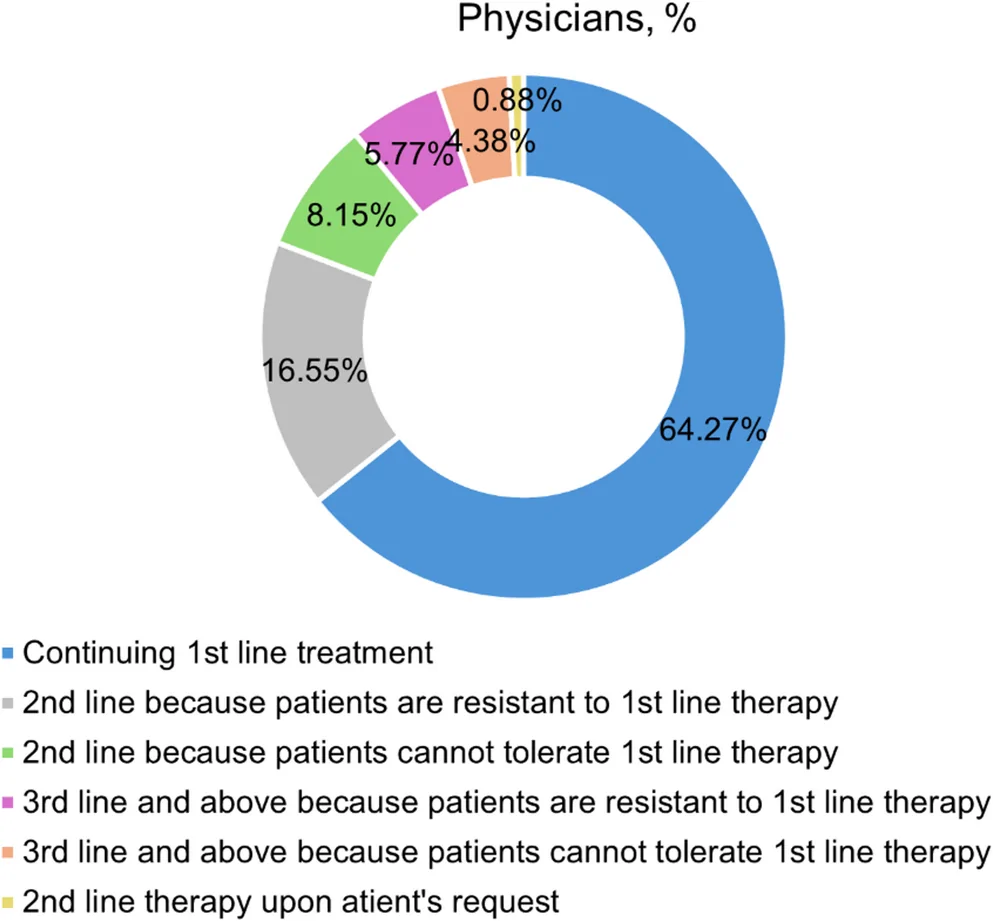
Physician-reported distribution of treatment lines among patients with CML. Data are based on physician-reported estimates from responding hematologists (*n* = 104)

Physicians (*n* = 104) reported predictions for the distribution of their current CML patients among various lines of treatment. As shown in Figs. 2 and 64.27% of patients still receive first-line TKI therapy. Resistance to first-line treatment was identified as the leading reason (16.55%), followed by intolerance (8.15%). For patients progressing to third-line therapy or above, resistance to second-line treatment (5.77%) remained a more common driver than intolerance (4.38%). Transitions made simply on patient requests were found to be modest (0.88%).

Efficacy was the major expectation from treatment, followed by the manageability of side effects and overall survival (Supplementary Table S1). The primary treatment goals in the first-line context were deep molecular response (80%) and major molecular response (78%). Major molecular response continued to be the primary objective in the second- and third-line setting.

A total of 94% of physicians reported sharing treatment goals with their patients. Physicians responding to this question (*n* = 91) indicated that treatment adherence did not vary between treatment lines in 52% of patients, increased in 38% from the second-line treatment onwards, and decreased in 10% (*n* = 91). According to physicians, patients skipped a median of 3 TKI doses per month (Q1–Q3: 2–5), and 80% of physicians reported that non-adherent patients informed them about their adherence issues.

Moreover, 91% of physicians believed that they had good communication with their patients. The majority of physicians agreed that patients were thankful for the chance to receive treatment (median score: 6 [Q1–Q3: 5–6]) and were more concerned about their health after receiving a diagnosis (median: 5 [5–6]) (Supplementary Table S2). On the other hand, opinions on the disease’s psychosocial effects were more inconsistent among physicians. Most physicians disagreed that patients avoid travel and social events (median: 3 [2–4]).

The surveyed patients (53% male; median age 54 years [quartiles 1–3 (Q1-Q3): 42–63 years]) and the subgroup of hematologists (*n* = 15) who treated these patients agreed on the top five pieces of information that should be provided to patients at treatment initiation (Table 3). Patients primarily prioritized understanding the course of CML, its impact on daily life, and their treatment, whereas safety, monitoring & follow-up were of greater importance to physicians. While effectiveness was the top priority for patients (41%) during treatment switch, safety was the main priority for physicians (47%).

**Table 3:** Information needs at the time of diagnosis and priorities during treatment switch

At the time of diagnosis Patients should receive about… ^a^	Physician opinion, *n* (%) *N* = 15	Patient opinion, *n* (%) *N* = 120
The prescribed medication (instructions for use)	14 (93)	117 (98)
The course of CML/ and the impact of CML on daily life	13 (87)	117 (98)
Monitoring and follow-up	15 (100)	110 (92)
Potential side effects of the prescribed medication	15 (100)	106 (88)
Clinical conditions/diseases that may affect therapy	12 (80)	82 (68)
Top consideration during treatment switch	***(N = 15)*** ***n (%)***	(N = 71)* ***n (%)***
Treatment effectiveness (stopping/slowing disease progression)	4 (27)	29 (41)
Management of side effects	7 (47)	27 (38)
Rapid onset of therapeutic effect	4 (27)	14 (20)

^a^ These are the frequencies of mention for the top five items

* Number of patients who underwent a treatment switch

**Fig. 3:**
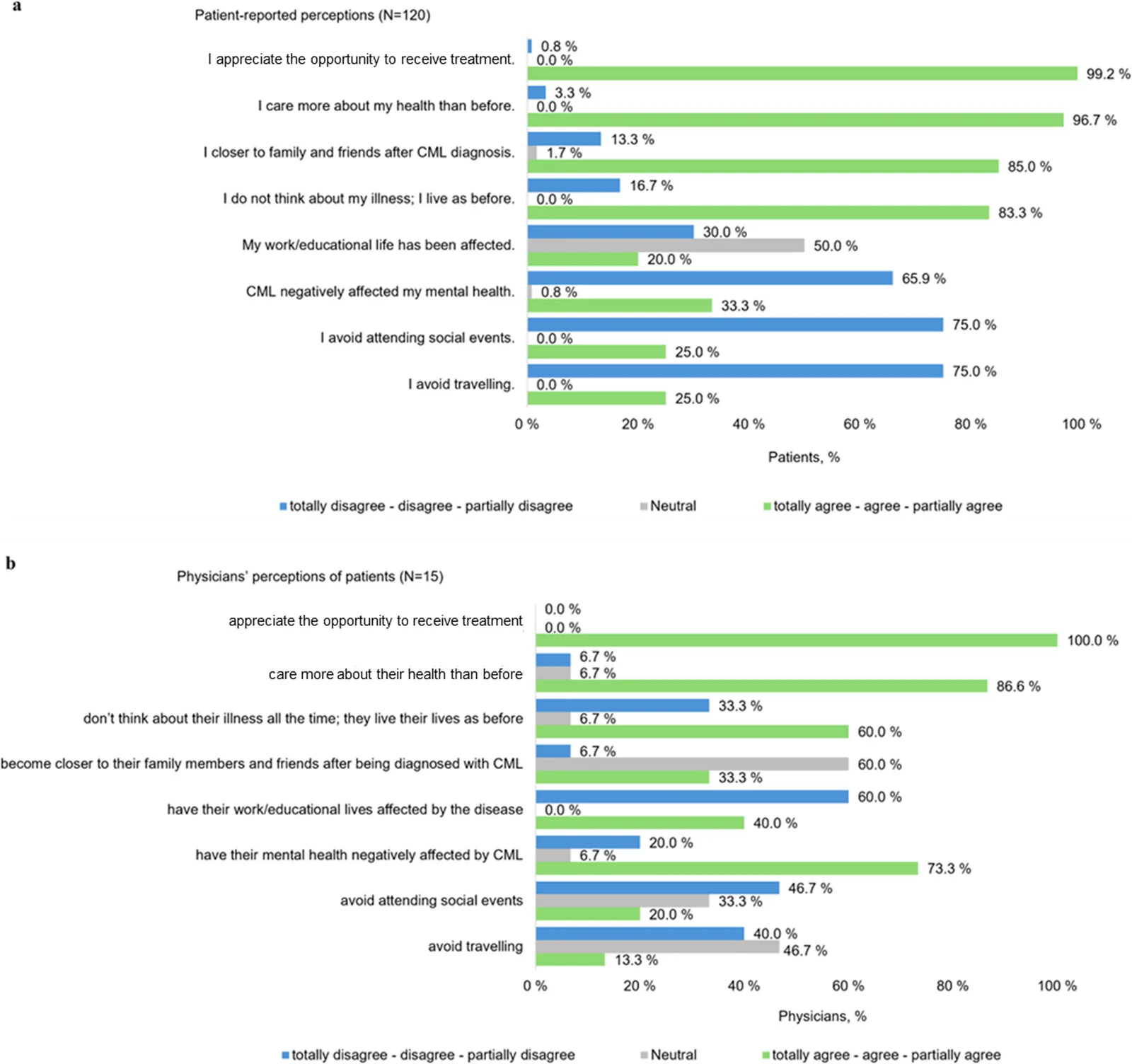
Patient and physician views on the impact of CML on patients’ lives (**A**) Physician-reported perspective (**B**) Patient-reported perspective. Responses were assessed using a 7-point Likert scale (1 = strongly disagree; 7 = strongly agree). Patient responses were based on 120 surveyed patients, and physician responses were based on the subgroup of 15 participating hematologists managing these patients

The perspectives of both patients and physicians regarding the effect of CML-CP on everyday living are demonstrated in Fig. 3, which includes 120 patients managed by 15 participating physicians from multiple centers across Türkiye. Both patients and physicians indicated a consensus that patients expressed gratitude for the chance to undergo therapy, and the patients became more concerned about their health after diagnosis. Physicians more often indicated that patients’ occupational or educational experiences were adversely affected and that mental health suffered (Fig. 3a), whereas patients expressed lower levels of agreement with these statements (Fig. 3b). Physicians indicated more consensus than patients over the avoidance of social gatherings and travel.

Among patients reporting nonadherence (*n* = 25), the median number of missed treatment days per month was reported as 3 (Q1–Q3: 1–4, *n* = 25) by patients and 2 (Q1–Q3: 2–5, *n* = 11) by physicians. Forgetting to take the TKI was the most common reason for nonadherence (patients: 52%; physicians:73.3%). Among these patients, 80% reported informing their physicians about missed doses or interruptions, while 86.7% of hematologists confirmed receiving such information from their patients. According to 56% of hematologists and 62.5% of patients, prior nonadherence and switching between TKIs did not affect treatment adherence. Anxiety or fear associated with TKI switching was observed in 32% of patients and 24% of physicians, while 35% of patients and 27% of physicians considered the treatment switch acceptable. Both groups reported follow-up visit frequencies of every 2–3 months, with a median visit duration of 10 min (Q1–Q3: 7–15).

## Discussion

This national survey study with 120 CML-CP patients and 129 hematologists from 33 institutions throughout Türkiye provides an overview of treatment expectations, need for information, TKI switching experiences, adherence patterns, and the reported psychological effect of CML-CP. Overall, the data suggest that disease management is still the most significant priority for both patients and physicians, but long-term tolerability, adherence support, and patient-physician communication continue to influence real-world treatment. These findings are consistent with the new CML-CP paradigm, in which treatment aims include obtaining and maintaining profound molecular responses as well as TFR for selected patients under strict monitoring [7, 8].

In this study, patients prioritized disease management and survival, while physicians concentrated on molecular response goals, safety, and monitoring strategies. International recommendations acknowledge that objectives have progressed from survival to include significant responses and, for eligible patients, TFR; however, achieving these objectives requires ongoing adherence, reliable monitoring, and patient-focused communication [7]. Recent patient-centered research indicates that objectives and expectations are situational and may vary across health systems and contexts [9, 10]. This underscores the potential importance of country-specific data in understanding patient expectations and treatment goals.

Most patients indicated that they understood the information given at diagnosis; however, only a limited number of physicians stated that patients completely understood the information. This variation might be clinically significant since CML management requires comprehension of response stages, monitoring protocols, and the balance between effectiveness and tolerability. The extensive patient-reported outcomes (PROs) literature suggests that clinician-based assessments alone may inadequately encapsulate the patient experience that contemporary methodologies support the integration of PROs and patient-centered toxicity/impact assessments to more accurately represent the lived burden and facilitate decision-making [11, 12].

The significance of comorbidity-informed TKI selection and long-term tolerability in regular treatment is highlighted by the median age (54 years), as well as the prevalence of diabetes and cardiovascular disease. Turkish real-world studies have also shown a significant burden of comorbidity and diverse practice patterns, which emphasize the need for tailored care in Türkiye [3, 4]. A minority of patients suffered temporary job reduction or early retirement after diagnosis, despite the majority reporting steady working status. This suggests that despite continued clinical management, some patients still have difficulties with daily or occupational functioning.

One-fourth of patients indicated missing doses, averaging 3 days per month, mostly attributable to forgetfulness. This trend corresponds with the ADAGIO research, which revealed that nonadherence is prevalent in standard treatment and is often motivated by unintentional factors such as forgetfulness [13]. Previous studies indicate that inadequate adherence might be a primary cause of failure to obtain adequate molecular responses in individuals anticipated to react favorably to imatinib [14]. In our study, the majority of non-adherent patients said that they informed their physicians, and physicians mostly acknowledged receiving this information. This may reflect a promising open communication between patient and physician that may promote prompt adherence, counseling, and tailored supportive treatments.

TKI switching often occurred due to adverse events and treatment inefficacy, which is consistent with real-world studies indicating treatment switching is prevalent in standard practice [2]. Patients appeared to prioritize disease control, while physicians focused on safety and the management of adverse effects during treatment switching decision-making. This distinction aligns with prior qualitative data indicating that decision-making requirements in CML often emphasize achieving a compromise between long-term disease management and the burden of prolonged therapy, particularly when there are more alternatives [15]. The fear and anxiety experienced throughout TKI switching in a significant proportion of patients suggest that enhanced communication and counseling during treatment transitions may be beneficial. In line with our results, previous research shows the psychological effects of treatment changes and suggests that patients may experience anxiety and worry during TKI switching [16].

Patients reported little impact on mental health and social engagement; conversely, physicians believed patients have a greater psychosocial burden and absenteeism. Prior data demonstrate that patient- and physician-reported assessments may markedly diverge, supporting the consistent incorporation of patient-reported evaluations to avoid systematic misestimation [17]. Moreover, negative medication experiences and treatment constraints have been linked to poor patient-reported outcomes and satisfaction in CML, highlighting the need for ongoing symptoms and tolerability evaluations, even within optimistic survival expectations [18]. Although the majority of patients reported never receiving psychological support, the underlying reasons for this finding could not be determined within the scope of this survey. These reasons may be attributed to variations in patient preference, symptom burden, psychosocial assessment practices, or access to supportive services.

Our findings suggest that CML-CP management in Türkiye largely aligns with internationally acknowledged priorities, while also highlighting divergence between physician and patient perceptions of comprehension, treatment transition, and psychosocial impact. These findings may also suggest the enhancement of shared decision-making structures by simplifying monitoring explanations, offering textual or visual choice assistance, assessing adherence, and providing organized switch counseling. Additionally, incorporating PROs beyond laboratory standards by consistently recording PROs, which can help align therapeutic expectations more closely with patients’ real experiences.

This study should be interpreted in light of several limitations. As data were gathered at a singular time point, responses may not accurately depict participants’ views throughout the disease trajectory. For instance, responses regarding diagnosis-related information needs or prior treatment decisions may be impacted by hindsight and experiences over years of follow-up. Secondly, participation relied on the willingness to complete the questionnaire, which can lead to selection biases. Physicians who answer via the Society platform and patients who consented to telephone interviews may vary from non-participants in their involvement with care, communication comfort, or motivation to share experiences. Furthermore, the identification and referral of patients by participating hematologists in routine clinical practice may have led to an overrepresentation of patients with stronger physician relationships, greater engagement in care or willingness to communicate. This potential should be taken into account when interpreting the relatively high levels of reported treatment comprehension, communication comfort, and disclosure of adherence difficulties, particularly regarding the generalizability of the findings. Also, the physician–patient comparison was performed at the group level instead of through direct pairing, potentially limiting the interpretation of concordance between individual patients and their treating physicians. In addition, several outcomes (e.g., missed doses, psychosocial effects) depended on self-reporting or physicians’ perceptions instead of objective metrics or validated instruments, potentially influenced by memory bias and social desirability. Despite these limitations, this study presents one of the most thorough dual perspectives assessments of CML management in Türkiye.

## Conclusion

Patients with CML-CP and their hematologists largely shared priorities and expectations regarding CML management. Patients often reported that their daily lives were more favorable than physicians assumed, suggesting some degree of discrepancies in psychosocial assessments. The frequency of follow-up visits aligns well with guideline recommendations, but visit durations are relatively short, which may limit addressing informational gaps, understanding the impact of CML on patients, and promoting collaborative decision-making. This may be particularly important in cases when anxiety is high, and barriers to adhering to the treatment plans are mostly unintentional. More structured communication at important decision points and adding low-burden adherence and psychosocial screening methods may facilitate the alignment of expectations and support patient-centered CML-CP care.

## Supplementary Information

Supplementary Material 1.

## Data Availability

No datasets were generated or analysed during the current study.
