# Supplementary material for: A survey on the management of chronic myeloid leukemia in chronic phase from the perspectives of both patients and physicians
Source: Ann Hematol. 2026 Jun 16;105(9):395. doi: 10.1007/s00277-026-07103-z (PMC13518523; doi:10.1007/s00277-026-07103-z)
Supplement: Supplementary file 1 [file 277_2026_7103_MOESM1_ESM.docx]

**Supplementary Material**

For the article “A survey on the management of chronic myeloid leukemia in chronic phase (CML-CP) from the perspectives of both patients and physicians”

**Table S1.** Physicians’ treatment goals at various treatment lines for CML-CP*

| **First-line**  **(n=112)** | **Second-line**  **(n=109)** | **Third-line and beyond**  **(n=107)** |
| --- | --- | --- |
| Deep molecular response (80%) | Major molecular response (74%) | Major molecular response (76%) |
| Major molecular response (78%) | Deep molecular response (67%) | Patient survival (56%) |
| Hematological response (60%) | Manageable side effects (60%) | Deep molecular response (54%) |
| Complete cytogenetic response (56%) | Patient survival (45%) | Manageable side effects (53%) |
| Manageable side effects (48%) | Hematological response (44%) | Reduce risk of serious adverse events (50%) |

* Proportion of mentions among the top five items.

**Table S2.** Physicians’ perceptions of the impact of CML on patients’ lives (n=103).

| Strongly disagree (1)  Disagree (2)  Partially agree (3) | Neutral (4) | | Somewhat agree (5)  Agree (6)  Strongly agree (7) | |
| --- | --- | --- | --- | --- |
| Patients with CML-CP… | **Physicians**  **n (%)** | | | **Median**  **(Q1-Q3)** |
| are grateful for the opportunity to receive treatment. | 2 (1.9) | 6 (5.8) | 95 (92.3) | 6 (5-6) |
| care more about my health than before. | 5 (4.8) | 8 (7.8) | 90 (87.4) | 5 (5-6) |
| don’t think about their illness all the time; they live their lives as before. | 52 (50.5) | 9 (8.7) | 42 (40.8) | 3 (3-5) |
| become closer to their family members and friends after being diagnosed with CML. | 19 (18.5) | 44 (42.7) | 40 (38.8) | 4 (4-5) |
| have their work/educational lives affected by the disease. | 58 (56.3) | 8 (7.8) | 37 (35.9) | 3 (3-5) |
| have their mental health negatively affected by CML | 35 (34.0) | 9 (8.7) | 59 (57.3) | 5 (3-5) |
| avoid attending social events. | 59 (57.3) | 22 (21.4) | 22 (21.4) | 3 (2-4) |
| avoid travelling. | 56 (54.4) | 23 (22.3) | 24 (23.3) | 3 (2-4) |
| CML-CP: Chronic Phase Chronic Myeloid Leukemia; Q1: first quarter; Q3: third quarter | | | | |
